# Early History of Practical Anatomy

**Published:** 1875-12

**Authors:** Wm. Clendenin

**Affiliations:** Professor of Anatomy in the Ohio College of Dental Surgery


### EARLY HISTORY OF PRACTICAL ANATOMY.

*An Introductory Lecture delivered by Wm. Clendenin, M. D., Professor of Anatomy in the Ohio College of Dental Surgery Oct. 13th, 1875.*

In our text-books on anatomy of the present day we find that the names of persons are, in many instances, given to certain parts of the body. In view of which fact I have thought it might be advantageous to you, young gentlemen, at the beginning of our course, to take a brief survey of the early history of anatomy, so that you may know who these persons were and what they did as laborers in the field of science to entitle them to the distinction thus given to them.

The early history of medicine consists largely of uncertain traditions, often mingled with fables; yet the oft-quoted statement of Pliny is undoubtedly correct: That if there exists any nation, which, at any epoch of its history, was without physicians, there is not one in which we do not find some vestiges of medicine. To be sure the Romans, who occupied themselves with killing, were satisfied to remain five hundred years without a regular physician. The Egyptian nation was, in medicine, as she was in other sciences, the instructress of the human race. Houdart gives special prominence to the fact that Egypt was the cradle of medicine, and in summing up his testimony of the immense knowledge of the savans of ancient Egypt, he indicates the titles of forty-two volumes of their writings, "six of which treated of medicine." We certainly know that at the time of the death of patriarch Jacob, seventeen hundred years before Christ, Egypt had her practicing physicians. We read in a book of Genesis that when Jacob died, "Joseph commanded his servants, the *physicians*, to embalm him; and the physicians embalmed Israel, and forty days were fulfilled for him, for so are fulfilled the days of those that are embalmed." Some have supposed that the practice of embalming, which was practiced at a very early day, served to familiarize the Egyptian priests of that period with the anatomy of the human body; but, according to Herodotus and other historians, this process was conducted in such a rude manner that it could not have contributed to the advancement of science.

Hippocrates—born four hundred and sixty years before Christ--a contempory of Socrates, of the famous age of Pericles, is familiarly spoken of as the "Father of Medicine." While the writings of Hippocrates, form the most ancient authentic monument in medical science, exhibit no traces of anatomical or physiological knowledge, yet he speaks of "the glands, as spongy viscera, destined to secrete humidity from the surrounding parts, and that the brain, the largest of the glands, attracts the vapors of all the interior of the body. The muscles were for the purpose of covering the bones; the nerves, the tendons, the ligaments, the membranes, are all represented as analogous organs, concurring in the same manner to the production of motion." His writings treat, in general terms, only of the form, volume, and position of the principal organs. He dissected only the bodies of the inferior animals. Some writers, indeed, deny that he even dissected animals, or that he ever had in his possession a human skeleton. So that we may conclude with the learned and candid Le Clerk, that the knowledge which Hippocrates possessed of anatomy was little, if at all, superior to that of his contemporaries.

It is to the munifience of the Ptolemies, who, about three hundred and twenty years before the Christian era, laid the foundation of the celebrated Alexandrian library and of the school of philosophy, which is graced with so many illustrious names, that we must ascribe a new era in the history of medicine. Ptolemy Soter, and his son and successor, Ptolemy Philadelphus, brought to Alexandria the most learned men of their times, gave them apartments in the museum, and created a revenue for their maintenance. The science of medicine was cultivated in this school with peculiar assiduity, and we owe some very essential improvements to its professors. Among the most famous of these were Erasistratus and Herophilus. History furnishes but little relating to the personal of these two individuals; but through Galen, Coelius, and Aurelianus we have a full account of their opinions and practice. They are particularly mentioned as being the first who dissected the human body. The school of medicine thus established at Alexandria eclipsed all its predecessors, and for several centuries it had no rival. In the time of Galen—two hundred years after Christ—so famous was it that it sufficed to have studied in Alexandria, or even to have resided there for a time, to obtain the reputation of a physician.

The success with which the healing art was taught at the Alexandrian school was chiefly due to the fact that its founders authorized the dissection of the human subject.

As I have already stated, Erasistratus and Herophilus were the first to take advantage of the unique authority thus granted—they were the first human anatomists of which we have any knowledge; and they amply profited by the advantage which was thus given them. They advanced our knowledge of the structure of the body, especially by pointing out the difference between the organs of the human body and those of the animals most nearly resembling it. Nearly every part of the great system of which the body is composed profited by their labors. The fame of the two anatomists is so intimately blended that it is perhaps impossible to assign to each his respective share of merit; but Herophilus was considered the most skillful in the practical department.

The practice of dissections did not long continue even in the city where it had its origin; yet the school of Alexandria produced a succession of learned men, in medicine, also in the other sciences. During the period covering the rise of the Alexandrian school, the Romans gave their attention almost exclusively to war-like affairs, so that science of all kinds, including medicine, was almost totally neglected; yet it was during this same period that she laid the foundation for her future greatness—she extended her empire beyond Italy to Egypt. Julius Cæsar burned the great Alexandrian library, a loss which Cleopatra sought in vain to repair through her spouse, Mark Anthony. But Roman domination was the scourge which proved destructive to the progress of medical science in Egypt. "That royal people, who delighted to see blood flow, not only on the battle-field, but also in their diversions and daily exhibitions, regarded as a profanation the contact of a corpse."

About the year 200 of the Christian era, we find in Rome one of those extraordinary characters who are destined to form an era in the history of science, both from the actual improvement which they have introduced into it, and from the ascendency which their genius enabled them to acquire over the minds of their contemporaries. That man was Claudius Galen. Galen was a native of the city of Pergamos, in Asia Minor, where there was a celebrated temple dedicated to Æsculapius. Galen enjoyed, both from birth and from education, every natural, acquired advantage. He made several voyages for instruction, and spent considerable time in Alexandria. His writings were very voluminous, amounting in all to about two hundred distinct treatises. He wrote a monograph on the human skeleton. in which he recommends that the bones be not studied in books only, but that they be seen and handled; and to do that, he advises the student to go to Alexandria, where he can see the human skeleton.

Galen undoubtedly had a knowledge of the bones composing the skeleton; and he was perhaps the first anatomist to teach the mechanism of locomotion, and to prove that the muscles take an active part in it. He says that the muscles are so numerous that they can not be easily counted, and that they unite in such a manner that several seem to form but one, and when they divide, there appears to be as many as there are tendons.

Galen's knowledge of anatomy was perhaps entirely limited to that derived from dissecting the ape and lower animals; yet he refuted the opinion that the arteries contained air, and not blood, and it must be admitted that in many respects, his works possess great merit.

But the zeal for dissection of the human subject was speedily dissipated. The Romans burned their dead, and the Koran of Mohammed prohibited even the touch of the corpse.

Rhazes, one of the most illustrious physicians of the Arabian school, who was born seven hundred years after Galen's time, paid no attention whatever to anatomy. The same may be said of Avicena, Ali Abbas, Meuse, and others of the Arabian school.

In the Saracenic school of Spain, which extended from the eighth to the twelfth century, the science of medicine seemed to retrograde, and practical anatomy was unheard of. From the twelfth to the fifteenth century, an interval of three hundred years, during which what are termed the dark agas still remain enveloped in the deepest gloom, every department of science was neglected, and among others that of medicine fell into the lowest state of degradation. The healing art, such as it was at that time, was in the hands of the monks, who still adhered to the doctrines of Galen, but with these they mixed up a large portion of superstition, and held not unfrequent recourse to magic and astrology.

Even as late as the fourteenth century, it was the custom to demonstrate anatomy on hogs and other animals, the organs of which were supposed to most nearly resemble the human body, making up the deficiencies by supposed analogies, or rather by the efforts of the imagination.

In 1315, Mondini, a professor in the university at Bologna, (in Italy), so far overcame vulgar prejudice as to have dissected two female subjects, and subsequently published a description of the human body, which appears to have had the rare merit of being drawn immediately from nature, and for a long time it was used as a text-book in many of the Italian universities. Mondini is also entitled to the credit of having given a very early, if not the first example of anatomical plates. But such was the prejudice against the dissection of the body that for more than one hundred years afterward no one dared to repeat the acts of the Bolognese professor. Even Mondini himself was not free from the thralldom of superstition, and he was not willing to open the head for fear of committing a mortal sin.

Thus, with a very few exceptions, during a space of more than a thousand years from the death of Galen, very little advance had been made in our acquaintance with the structure of the body. The professors of the Arabian school, with their successors in Italy and France, for the most part contented themselves with copying the descriptions of the ancients, without ever calling in question their accuracy. Even after the examination of the human body had become more common, it was long before the profession could so far free themselves from the tyranny of authority as to admit that any imperfection could exist in the works of Galen, and the researches of all the anatomists named were made in accordance with his teachings.

It was reserved for Andrew Vesalius to correct the errors of Galen; and this he was enabled to do successfully, because, as we have already said, Galen's descriptions were, for the most part, made from dissections of apes, and did not therefore correctly represent the conformation of the human body.

Vesalius was born in Brussels, in the year 1514, of a family long illustrious in medicine. He was the first anatomist who threw off the yoke of authority, which had been imposed by a blind veneration for the opinions of the ancients.

Renouard (History of Medicine) gives the following version of the story as to the way Vesalius obtained his first skeleton: "Having observed the body of a criminal, of which the birds had so perfectly cleaned away the soft parts that there remained of it only the bones and ligaments, he detached successfully the extremities; but when he attempted to carry off the trunk, he found it so strongly bound to the stake by iron chains that he was compelled to work all night to get it loose." We find our hero next in Paris, in his zeal to observe nature for himself, disputing with the dogs and vultures for the remains of criminals. In his twentieth year he discovered and demonstrated the semilunar valves of the aorta. "At the age of twenty-three, he was nominated to the chair of anatomy in the faculty at Padua, by the Senate of Venice; at twenty-nine (in 1543), he published his great work, in which this science is placed in a new light, and with a completeness which left far in the rear all that antiquity had transmitted on the subject." In the following year he was called, by the emperor Charles V., to the court of Madrid, then the most brilliant in Europe, in the character of first physician, after which he abandoned forever his anatomical studies.

Long and bitter discussion occurred between the defenders and opposers of Galen's teachings, and it was not until after many years of severe struggle that the truth was established, and that it was finally admitted that the errors which had been pointed out by Vesalius actually existed.

Anatomists everywhere followed Vesalius, among whom were some of those who had defended the teachings of Galen: Eustachius, professor of anatomy at Rome; Fallopius, a pupil of Vesalius, professor at Padua and Pisa: Columbus, a friend and pupil of Vesalius, and his successor to the chair of anatomy at Padua. And I may mention also the names of Patricius, Gassen, Vidius, Arantius, Varolius, and others whose names are so intimately connected with the study of anatomy.

The prejudice against dissecting the human subject remained unabated, and was strengthened by papal bulls, by royal decrees. But at the period of which we are speaking a grand political revolution was commencing in Europe, which eventually produced an entire change in the civil conditions of its inhabitants, and indirectly affected, in an equal degree, its sciences and its literature. The effects of the crusades of the Reformation, and of the invention of printing "an art which derides the havoc of time and barbarism," was to give a new impulse to learning and the arts, and particularly to medicine. Public dissections were made in the universities of Italy. Bologna, which had been the first, continued to be one of the most celebrated schools of anatomy, and her fame was heightened by the names of Vesalius, Malpighi, Valsalva, Varolius, and others of equal distinction, and, in connection with one of the great social questions of the day, it may be of interest to you to know that this list of savans was graced by a woman—Madonna Manzolina—who was among the most distinguished professors of anatomy at Bologna.

The influence of the great men just named was felt and acknowledged by the more intelligent everywhere, and even by the Roman pontiff and kings. Up to this period, dissections were made by the teachers of anatomy only, and this was done in some private chamber, but under no circumstanees were students permitted to handle the scalpel or *razor* for themselves, for the latter instrument was the one in most common use. Anatomical chairs were soon afterward created; amphitheaters were erccted and provided with facilities for dissections; more liberal laws were enacted, rendering dissecting material more abundant, and less difficult and less hazardous to obtain.

Under these favoring circumstances many important discoveries were made. Fabricius discovered the valves in the veins; the pulmonary circulation was explained by Columbus and Cesalpine; Michael Servetus, who was burned at the stake by Calvin, discovered how the blood was conveyed from the right to the left side of the heart, and, indeed, Cesalpine almost attained a full conception of the circulation of the blood. The nerves were entirely separated from the tendons and some idea was had of the lymphatic system. Thus anatomy began to grow into importance, and came to be looked upon favorably by the profession at large. Among the distinguishing features of this period (the sixteenth century) is to be noted the fact that in the various universities which were established in Southern Europe, anatomy and other branches of medicine began to form a very distinguished part of the teaching. First in the order of time was the University of Salerno—first after the destruction of the Roman empire; the second, that of Montpelier. The University of Bologna is said to have had some celebrity as a school of medicine as early as the thirteenth century. Subsequently, medicine was taught in the universities of Vienna, Paris, and about half a century afterward, medical schools were established in Padua, Pavia, Milan, Rome and Naples. To these schools hundreds of students were convened from all parts of Europe.

England was the first to profit by the brilliant example of Italy. Through the efforts of Dr. Cains, the College of Surgeons was founded in 1540; and, in 1581, the College of Physicians created the lectureship on anatomy, and two years afterward built the Knight River Street Anatomical Museum, the first constructed in Great Britain. It was in this amphitheater that Harvey gave his first demonstrations of the circulation of the blood about the year 1617, but his first publication of his great discovery was not made until several years later.

But before noting the life and labors of Harvey, it may be proper to recall what were the acquisitions of his predecessors. The liver was considered, from time immemorial, as the organ of sanguification. It was supposed that the veins took their origin in the liver, and that they were the sole order of vessels that contained the blood. The arteries were supposed to contain, in their normal state, vital spirits only of which the heart was the great reservoir. Galen modified this doctrine by demonstrating that the arteries contained blood at every period of life, but he supposed that the blood did not flow through the lungs, but that it reached the left ventricle by passing through the porosities of the septum. The opinion was not contested until the middle of the sixteenth century. At that epoch, the theologian, Michael Servetus—the same who perished, a victim of the jealousy of Calvin—dared to deny the passage of the blood through the septum of the ventricles, and contended that the blood flowed through the lungs and thence returned to the left side of the heart. Fabricius had discovered the valves in the veins. Such was the state of science at the beginning of the seventeenth century. There was only one step to take to find the true course of the blood, but that step was difficult, as we may now readily understand. This step was taken by, and has immortalized the name of William Harvey.

Harvey was a native of Folkstive, county of Kent, England. Having studied at home, he subsequently went to Italy, and remained at Padua four years attending the lectures of Fabricius of Aquapendente. He returned to his own country with the title of doctor, in 1602, and established himself in London and became regent of the College of Medicine, in 1613; about that time he commenced to make known his doctrine of the circulation of the blood in his lectures, but he did not publish the results of his researches till 1628. I briefly give you his own story, in which he depicts the obstacles he met with in his efforts to discover the truth: "Devoting myself to discern the use and utility of the movements of the heart in animals in a great number of vivisections, I found at first the subject so full of difficulties that I thought for a long time, as Francaster, that the secret was known to God alone. I could distinguish neither in what manner the systole and diastole took place, nor at what moment the dilation and constriction occurred, owing to the celerity of the movements of the heart, which, in most animals, is executed in the twinkling of an eye or like the flash of lightning. I floated, undecided, without knowing on what opinion to rest. Finally, from redoubled care and attention, by multiplying and varying my experiments, and by comparing the various results, I believed I had put my finger on the truth and commenced unraveling the labyrinth. I believed I had seized the correct idea of the movement of the heart and arteries, as well as their true use."

Renouard remarks that, "So much care and circumspection in the search for truth, so much modesty and firmness in his demonstration, so much clearness and method in the development of his ideas, should have prepossessed every one to favor the theory of Harvey; but, on the contrary, it caused a general stupefaction in the medical world, and gave rise to much opposition. This theory, which appears to us to-day so natural that we conceive with difficulty why it was not found much sooner, was nothing less than a revolution in physiology. The controversy it excited lasted over a quarter of a century, and there was not a man at the time who made any pretension to a knowledge of anatomy, who did not take an active part in it. Even the naturalists and philosophers did not remain indifferent." Harvey, however, had the satisfaction, before his death, to see his theory of the movements of the blood universally adopted.

While we have said that England was the first to profit by the example Italy afforded, it is probably more correct to give this honor to Holland. Ruysch, Swanmerdam, Albinus and Boerhaave were the great anatomists of Holland. Haller, the great German anatomist, studied in Holland, and it was here, too, that the Monros, of Scotland commenced their anatomical course, which subsequently made them so famous in their own country. Next, in point of time, Denmark, Sweden, then Germany, France, and England, became distinguished for their schools of anatomy.

"The first Scotch anatomical museum was built (*Keen's Lectures* on the Early History of Practical Anatomy), and the first public demonstration given, in 1697. But it was not till 1720 that a regular professor was appointed. At that date Monro the first was elected professor at the extraordinary salary of fifteen pounds per annum. From this time till 1859, when Monro the third died, the history of Edinburg anatomy, and that of his astonishing family, are almost identical. True, John Bell and Knox, Charles Bell, Barclay, Innes, and others lectured in private schools; but the Monros held the scepter. All of them lived to old age—Alexander primus dying at seventy, Alexander secundus at eighty-four, Alexander tertius at eighty-six. All were professors early in life; at twenty-three, twenty-one, and twenty-five respectively. All of them taught for long periods—thirty-eight, fifty-four, and forty-eight years; and father, son, and grandson. they held the anatomical chair in Edinburg, from 1720 till 1846, a period of one hundred and twenty-six years."

Our brief sketch would be imperfect, did we fail to make one reference to the life and labors of Marie Frances Xavier Bichat, who was born November 11, 1771, and died at the early age of thirty-two. He was a pupil and adopted son of the great French surgeon, Desault. Bichat is the first anatomist, and to him belongs the credit of separating the human body into elementary tissues, and to ascertain the peculiar properties which characterize each tissue.

There is no science the study of which has always been attended with more embarrassments and difficulties than that of practical anatomy. At all times and everywhere, it has been generally regarded with popular odium; the highest degree of opprobrium has attached to the means and methods necessary for its cultivation, especially because bad men have been employed, and employed, too, in an illicit transaction—that is, aiding to make the dead subservient to the dearest interests of the living. The men here referred to have long been known as resurrectionists, or body-snatchers. Physicians know very well that it is wholly impossible for any one, in any branch of the profession, to become a competent practitioner, unless he be fully conversant with the healthy structure of the human body. There is but one way by which this knowledge can be obtained, and that is by the dissection of the dead. The most eminent men in the medical profession everywhere, and at all times, are those who are most distinguished for their knowledge of anatomy. The immediate obstacle, and the one attended with the greatest difficulties and annoyances has grown out of the want of material (dead bodies), or of the best means of obtaining it without doing vioence to the feelings of the community.

The first and only legal source of material was executed criminals. Henry VIII. gave the College of Surgeons the privilege of dissecting four felons annually; and Queen Elizabeth granted the College of Physicians the same privilege in 1564. During the reign of George II. of England, in 1726 *all* criminals were given over to the doctors for dissection. This act continued in force till 1832, when the well-known anatomy acts were passed, making a more liberal provision for obtaining dead bodies for dissection in the medical schools.

In this country, practical anatomy was first legalized by the legislature of Massachusetts, in 1831, and soon after by New York. With these exceptions, in our own country, the dissection of the dead is still unlawful. Our legislators have almost always been on the side of the superstitious masses, and they have generally answered the petitions of the physicians by passing the most stringent laws—even the bodies of criminals who have died on the scaffold to expiate the highest crime, have been protected from the touch of physicians, as if the dissections of their bodies would be a still greater crime than that for which they were executed.

The law virtually proclaimed, as it now does, "that the surgeon should possess aptitude and skill (as well as a diploma,) and subjected him who failed to display proper skill to pecuniary forfeiture, in the civil courts, at the instigation of any dissatisfied patient; yet the only mode of acquiring that skill—namely, from dissecting the dead clandestinely obtained—was, in the criminal courts, held to be a misdemeanor, punishable by fine and imprisonment." Such is, in effect, the law under which we in Ohio, are studying anatomy to-day; and such are the laws which, *for so long a time*, forced upon communities the services of resurrectionists and the crime of body-snatching.

To relate the many stories told illustrative of the character and adventures of these men—the resurrectionists—would require much more time than we are permitted to give the subject. I will give but one illustration. Those who wish to pursue the subject further, will find in the lives of Sir Astly Cooper and John Hunter full details. But to our illustration which I take from the life of Sir A. Cooper. A pupil, who was conveying a body, by a coach, to his hospital, was astonished to find himself in front of Bow-street police headquarters, when a driver, tapping at the front window of the carriage, said to his affrighted employer within: "Sir, my fare to so-and-so is a guinea, unless you wish to be put down here." The reply, without any hesitation was: "Quite right, my man; drive on." In order to protect the grave-yards, in the vicinity of London, the walls were sometimes raised six or eight feet above their usual height, and topped out with broken glass and iron spikes; spring guns were set in the church-yards, but they were useless, because if the resurrectionist was not intimate with the grave-digger, or watchman, he sent a woman to the funeral as a mourner, to note the position of the traps to which the wires were attached. Graves were often watched for weeks by persons hired for the purpose, and often, too, by relatives of the deceased, but all these devices and watchers were useless against the persistent efforts and cunning of the resurrectionists, as the high price for subjects—often as much as five hundred dollars for a single subject—was sufficient to induce them to incur all risks. So daring and expert were these men, and such their character, that Sir A. Cooper stated, in his evidence before the parliamentary committee, that no matter what the social position of any person in England, he could obtain his body if he desired it; and such villians were they, that for a respectable price, they would unhesitatingly make a subject of him, their best, though unwilling patron.

May we not, with truth, assert that governments ought to sustain, at least some degree of the odium which attaches to resurrectionists and anatomists?

